# Phyto- and Microbial-Based Remediation of Rare-Earth-Element-Polluted Soil

**DOI:** 10.3390/microorganisms13061282

**Published:** 2025-05-30

**Authors:** Wei Dong, Yuexin Song, Luyao Wang, Wenchao Jian, Qian Zhou

**Affiliations:** 1 Jiangxi Provincial Key Laboratory of Environmental Pollution Prevention and Control in Mining and Metallurgy, Ganzhou 341000, China; 2 School of Resources and Environmental Engineering, Jiangxi University of Science and Technology, Ganzhou 341000, China; 3 Yichun Lithium New Energy Industry Research Institute, Jiangxi University of Science and Technology, Yichun 336023, China; 4 School of Life Sciences, Jiangxi University of Science and Technology, Ganzhou 341000, China

**Keywords:** environmental pollution, bioremediation, plant–microbe interaction

## Abstract

Rare-earth elements (REEs) are strategic resources that have been extensively utilized in industrial manufacturing, aerospace engineering, and defense technology. Beyond their technological applications, REEs have been demonstrated to enhance agricultural productivity through growth promotion mechanisms in various crops, leading to their recognition as valuable trace element fertilizers in modern farming practices. Consequently, REEs have been increasingly introduced into ecosystems, where they are continuously accumulated in soil and transmitted into food chains, resulting in REE pollution, which has become a significant environmental concern. However, the regulatory mechanisms controlling REE contamination are not well understood. In recent years, the environmental impacts of REEs have attracted increasing attention, especially in their pollution mitigation from industrial and agricultural REE emissions. Bioremediation is regarded as a promising method for contaminated soil treatment. The application of plants and microorganisms to REE-polluted environments has been explored as an emerging research field that combines the synergistic advantages of plant rhizospheric microorganisms and vegetation systems. The combination of phytoremediation and microbial remediation approaches has been shown to enhance soil health restoration, thereby improving the purification efficiency of REE-contaminated soil. This paper, citing 179 references, reviews the roles of plants, microorganisms, and plant–microbe interactions in REE-contaminated soil remediation, and summarizes the available practical methods with which to address REE pollution and the fundamental mechanisms involved.

## 1. Introduction

Rare-earth elements (REEs), which encompass the lanthanide series, scandium (Sc), and yttrium (Y), are categorized into light rare-earth elements (LREEs) and heavy rare-earth elements (HREEs). LREEs include lanthanum (La), cerium (Ce), praseodymium (Pr), neodymium (Nd), samarium (Sm), promethium (Pm), and europium (Eu). HREEs include gadolinium (Gd), terbium (Tb), thulium (Tm), dysprosium (Dy), lutetium (Lu), erbium (Er), ytterbium (Yb), holmium (Ho), Sc, and Y [1,2,3]. The designation of “rare” is not universally applicable to all rare-earth elements. A representative example is Ce, the environmental abundance of which has been documented to be comparable to that of commonly occurring metallic elements such as copper and zinc [4,5]. REEs are usually found to combine with several elements and exist in the forms of phosphate minerals, carbonate minerals, and chlorides. They are dispersed in the accessory minerals within granite, pegmatite, and related common rocks [2,6,7,8]. However, environmental problems, especially those related to soil, can arise from both the mining and utilization of REEs. As one of the metallic elements, REEs can be transferred through activities such as mining, metal extraction from ores and minerals, metal smelting, medical facilities, and petroleum refining [9]. There are numerous methods for remediating metal-contaminated soils, including physical, chemical, and biological approaches.

Most physical remediation techniques feature simple equipment and easy operation, of which thermal desorption and soil replacement are typically used methods [10]. Thermal desorption separates pollutants from the soil through direct or indirect heating. It has advantages such as a short cycle, high efficiency, and high safety. However, it is limited by expensive equipment and a relatively long desorption time [11]. Soil replacement involves the replacement of deteriorated soil with safer soil to reduce the amount of chemical components in a specific area. Nevertheless, it requires a large amount of work and incurs high costs [12]. Chemical remediation methods mainly include chemical leaching, chemical stabilization, and chemical oxidation/reduction [10]. Chemical leaching is characterized by high efficiency, low costs, and simple operation [13]. However, it can lead to secondary environmental pollution and hinder the normal growth of plants [12]. While stabilization demonstrates technological viability in immobilizing contaminants through cost-efficient and operationally feasible methodologies [14], critical parameters including long-term stability performance, lifecycle cost–benefit ratios, and ecological side effects on soil matrices require systematic evaluation prior to field-scale implementation [15]. Through chemical oxidation or reduction, the valence states of heavy metals can be altered, thereby reducing their toxicity and achieving the objective of remediation [16]. However, chemical oxidation–reduction is sometimes limited by costs and the potential for secondary pollution [10].

**Figure 1:**
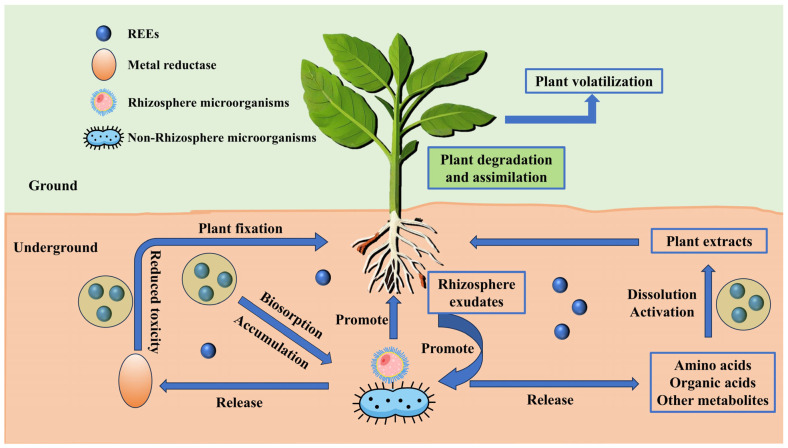
Remediation of REE-contaminated soil using the plant–microorganism combined technique.

In contrast, the bioremediation of REE-contaminated soils primarily utilizes the metabolic activities and physiological mechanisms of organisms (e.g., plants, microorganisms) to absorb, immobilize, or transform REEs in soil, thereby reducing their environmental risks. This approach is not only environmentally friendly but also economically advantageous as it leverages natural ecological cycles. Based on the primary remediation agents, the techniques can be classified into the following: phytoremediation, microbial remediation, and plant–microorganism combined remediation. Herein, we delve into the pollution of REEs in soil, exploring their interactions with plants and microorganisms. This paper focuses on understanding the mechanisms of plants and microorganisms in remediating contaminated soil, and developing plant–microorganism combined remediation technologies to remedy REE pollution. Figure 1 illustrates the dynamic processes of REEs in the soil–plant–microorganism system:

(i) REE Transformation: Metal reductases collaborate with rhizosphere/non-rhizosphere microorganisms to catalyze the transformation of REEs’ chemical forms, regulating their bioavailability.
(ii) Soil Layer Functions: Plants influence REE migration through degradation and fixation, while soil REEs either undergo toxicity reduction before release or diffuse via underground pathways.
(iii) Plant Fixation Mechanisms: Plants accumulate REEs via biosorption, secrete rhizosphere metabolites (e.g., organic acids) to dissolve and activate REEs, and ultimately balance their distribution and toxicity through extraction or release.
(iv) Plant Volatilization: As a core phytoremediation mechanism, plant volatilization not only directly drives the migration and transformation of pollutants within plants, but also enhances the rhizosphere environment and regulates microbial activity through systematic coordination.

## 2. Environmental Impact of REEs on Soil

REEs are recognized as indispensable components in modern industrial systems. Their critical importance has been progressively amplified through scientific and technological developments due to their distinct physicochemical properties. For instance, the fabrication of high-temperature superconductors requires the utilization of La and Y [17], while compounds containing Eu and Ce are extensively employed in the production of advanced magnetic materials [2,18]. Furthermore, Ce, La, and Y are utilized as critical catalytic components in industrial processes [19]. REEs have been applied across diverse technological domains including precision polishing systems, photoluminescent materials, renewable energy infrastructure (specifically electric vehicles and wind turbines), advanced electronics, agricultural enhancements, and biomedical innovations [20,21,22,23,24,25,26,27].

Studies have indicated that although REEs can accumulate within soil, they generally have low mobility [28,29]. The sources of REEs in soil can be divided into natural and anthropogenic sources. In the natural environment, REEs from some minerals enter the soil partially or entirely through weathering processes [30]. In addition, artificial mining of rare-earth ores can also lead to REE exposure and even potential environmental issues [31,32]. When cultivating crops, most REEs used as seed coatings or foliar fertilizers enter the soil. These elements can alter the physical and chemical properties of farmland soil surrounding mining areas, reduce soil fertility, and hinder the growth and development of crops [33,34]. REEs exhibit significantly higher solubility and reactivity when applied in fertilizer formulations compared to those naturally occurring in soil matrices. Consequently, their adverse environmental impacts are manifested more directly and rapidly [35]. However, the content of REEs is significantly higher than the national standard in soils that have been mined or are currently being exploited for rare-earth minerals [36]. Data indicate that REEs in soils surrounding the Bayan Obo deposit (Inner Mongolia, China) exhibit an average concentration of 4.67 × 10^3^ μg/g [37], while those near mining areas in southern Jiangxi range from 264 to 15,955 μg/g, both significantly exceeding the national background value (186 μg/g) [38]. And concentrations in soil or water near strong alkali sources may locally reach levels detrimental to local populations’ health [39]. Chronic exposure to REEs through oral or inhalation pathways has been linked to their progressive accumulation in human tissues, resulting in chronic toxicological effects like reductions in children’s cognitive performance, impairments to the central nervous system in adults, and elevated risks of cerebrovascular diseases [3,40].

## 3. Interaction Between REEs and Plants

**Figure 2:**
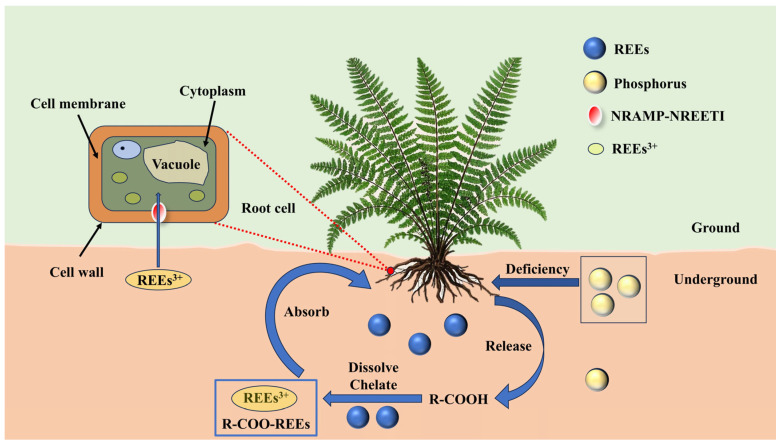
Mechanisms responsible for REE uptake and accumulation by plants.

REEs disrupt plant growth parameters by interfering with critical defense mechanisms, including osmoregulation through osmolyte synthesis, activation of antioxidative defense systems, modulation of secondary metabolic pathways, and phytohormone signaling networks [41]. Additionally, the concentration of REEs in plant tissues can be altered through plant accumulation. For instance, the levels of reactive oxygen species (ROS) and antioxidative enzyme activities in rice seedling roots were significantly elevated under Nd stress. Furthermore, Nd exhibited preferential accumulation in subcellular compartments, including soluble fractions, organelles, and cell wall structures [42]. REEs have been demonstrated to promote the production of crop biomass at low concentrations, while they inhibit crop growth at high concentrations [43,44,45,46]. The mechanisms associated with the uptake and accumulation of REEs are illustrated in Figure 2. Under phosphorus-deficient conditions, plants release organic acids to solubilize soil-bound phosphorus, while REEs and co-occurring metals may be inadvertently mobilized and absorbed by roots through collateral chemical interactions [47]. Organic acids such as malic acid and citric acid are found to enhance the absorption and accumulation of La in *Hordeum vulgare* L [48]. Studies have revealed that the Natural Resistance-Associated Macrophage Protein (NRAMP) family member NRAMP-NREET1, a root-specific transporter in *Dicranopteris linearis*, mediates the absorption of REEs from the root cell wall and their subsequent translocation into cytoplasmic compartments [49,50].

The application of appropriate doses of REEs to crops has been proven to enhance growth and yield by improving resistance to adverse growing conditions [51]. The observed beneficial effects may be attributed to the REE-mediated enhancement of nutrient phytoavailability and/or elevated chlorophyll biosynthesis through biostimulation mechanisms [46,52]. There are numerous reports on the distribution of REEs in various plant tissues [44,53,54]. For example, La can replace calcium (Ca) in plant structures and bind to cell walls, functioning as a second messenger [55]. Different concentrations of La, Y, and Ce significantly affect germination rates and root growth speeds in plants [56], with seedlings under high-concentration La stress exhibiting decreased growth and disrupted photosynthetic mechanisms [57]. Numerous studies have investigated the effects of REEs, such as La and Sc, on critical physiological parameters (e.g., growth parameters, chlorophyll content) in *Citrus* species and *Oryza sativa*. These findings consistently reveal a biphasic concentration-dependent response: low-dose REE exposure enhances plant performance through stimulatory hormesis, whereas elevated concentrations trigger inhibitory effects, disrupting metabolic homeostasis [58,59]. For example, compared to the edible portions of *Oryza sativa* and *Zea mays*, REE concentrations were found to be more highly increased in their roots and leaves [60]. At the same time, some REEs have been observed to exert a toxic effect on plants. The inhibition of phosphorus uptake has been identified as a primary toxicological manifestation of REEs in plant systems [44]. Once REEs are introduced into plants, ROS are generated, resulting in oxidative stress and plant cellular damage [61]. Prolonged exposure (90 days) of Zea to 25 mg/kg CeCl_3_ was observed to disrupt antioxidant system homeostasis, causing significant oxidative damage in root tissues through ROS overaccumulation [62]. Under hydroponic conditions, different forms of La exhibit marked toxic effects on the growth of broad beans [63]. Marques et al. found that the severity of leaf damage on soybean (*Glycine max*) exhibited a positive correlation with the tissue accumulation of Ce and La, while the foliar application of REEs did not significantly alter chlorophyll content [64].

## 4. Interaction Between REEs and Microorganisms

REEs not only affect plants and animals but also have an impact on the growth, reproduction, and metabolism of microorganisms [45]. In agriculture, REEs are typically used in combination with other essential trace elements, but their effects on soil microorganisms and subsequent impacts remain unclear. Several studies have reported that REE mixtures and La can affect the growth of certain bacteria and fungi [52,65,66]. Feeding experimental animals (piglets or broiler chickens) with REE-enriched feed may alter their primary fecal microbial communities [67]. REEs can enter bacterial cells through various passive and active uptake mechanisms, binding to the exterior surface of bacteria as well as penetrating into the cytoplasm [67,68,69]. It has been reported that the metabolism of *Escherichia coli* is slightly stimulated when it is treated with La at concentrations of 50 to 150 mg/mL. However, 400 mg/mL of La can cause damage to *E. coli* cells. The reason may be that the ionic radii of La and Ca are very close, and their ligand specificity is similar, allowing La to replace Ca at binding sites and disrupt cell membrane formation [70,71]. High concentrations of REE ions exhibit cellular-level inhibition in specific *Bacillus* species, contrasting with negligible effects on spores. Notably, both vegetative cells and spores can absorb rare-earth ions, suggesting their potential applications in bioremediation strategies for REE-contaminated environments [72,73]. However, there is a concern that the lysis of microbial cells or spore germination causes REE release into the surrounding environment [72].

REEs can affect the growth of fungi, beneficial fungi and soil-borne fungi [74], and can also be accumulated by fungi [75]. The fungus was found to have overall good tolerance to REEs when La alone or an REEs mixture was added to the growth medium for fungal culture, and the presence of appropriate concentrations of REEs in the growth environment of fungi may have a beneficial effect on their growth. For instance, the growth of *Trichoderma harzianum* T22 was enhanced under mixed REE treatments compared to single-element exposure, suggesting that the differential interaction mechanisms between an REE mixture and the fungal system depend on elemental composition ratios [65]. The amount of REEs accumulated by *Trichoderma atroviride* P1 was higher than that accumulated by *T. harzianum* T22 under the same conditions, which indicates that there is no direct correlation between REE-induced growth stimulation and the accumulation of REEs in fungal biomass [65]. Multiple studies demonstrate that fungi exhibit selective absorption capabilities for specific REEs, suggesting strategic applications in removing significant quantities of REEs from contaminated substrates during soil remediation through extracellular immobilization within fungal matrices [65,76]. Fungi can synthesize secondary metabolites, modulate enzyme activity, regulate metal-induced protein synthesis, and form complexes with REEs [77,78]. However, REEs were detected in the fungal cytoplasm, indicating that REEs can pass through the cell wall and cell membrane of live *Trichoderma* cells to reach the cytoplasm. Gao et al. demonstrated, for the first time, the presence of REEs absorbed by fungal cells [79]. REEs exhibit concentration-dependent dual effects (low-concentration stimulation and high-concentration inhibition) on soil microorganisms, and while their selective absorption by fungi offers potential pathways for bioremediation, the underlying mechanisms and ecological impacts still require in-depth exploration.

## 5. Bioremediation of REE-Contaminated Soil

Bioremediation employs biological entities like plants, animals, and microbial agents to degrade or sequester environmental contaminants through metabolic processes [80,81,82]. Soil bioremediation is achieved through contaminant extraction, transformation, and immobilization–degradation processes. Notably, while immobilization does not alter contaminant concentration, it reduces the environmental risk associated with contaminants by changing their mobility and bioavailability for living organisms [83,84,85,86]. Bioremediation is predominantly mediated through natural mechanisms, exhibiting low environmental perturbation. For instance, its reliance on natural processes minimizes environmental disruption, making it a cost-effective and eco-friendly solution for soil decontamination. Notably, *Pohlia flexuosa* moss demonstrates not only substantial La accumulation capabilities for extracting REEs from contaminated substrates but also exhibits exceptional preservation characteristics [87]. Bioremediation generally involves the in situ remediation of contaminated soil, which is time-consuming and highly dependent on environmental factors such as climate and the geological conditions of the remediation site [81,88]. Anthropogenic mineral processing activities inevitably induce metallic element dispersion, with REEs emerging as significant environmental contaminants. Therefore, strategies encompassing phytoremediation, microbial remediation, or a combined approach integrating plant and microorganism can be employed for contaminant sequestration in REE-contaminated soil.

### 5.1. Remediation of REE-Contaminated Soil by Plant

As a core bioremediation strategy, phytoremediation utilizes plant-mediated extraction mechanisms to sustainably eliminate contaminants from soils through phytoextraction, rhizofiltration, and phytodegradation processes [82,89]. This method proves particularly effective when the pollutant-affected area is extensive and the contaminants are within reach of plant roots [90]. Phytoremediation offers distinct ecological advantages including soil structure preservation, cost-effectiveness, and avoidance of secondary pollution [91]. This approach concurrently achieves enhanced vegetation coverage, ecological restoration in mining districts, and esthetic enhancement through landscape integration [92]. Furthermore, REE resource recovery is enabled through hyperaccumulator species in phytomining systems [93]. Phytoremediation efficacy is constrained by inherent botanical growth rates, necessitating extended temporal commitments for measurable contaminant reduction [94]. Implementation success is further modulated by edaphic and climatic parameters that govern plant viability [95]. The remediation of soils contaminated with rare-earth metals can be carried out through various phytoremediation mechanisms, such as phytoextraction, phytostabilization, phytovolatilization, and rhizosphere filtration [96]. Among these, phytoextraction and phytostabilization stand out as prevalent phytoremediation techniques for soils contaminated with potentially toxic elements, and are also applicable to soils contaminated with REEs [82]. Phytoextraction, recognized as an in situ and low-cost method, utilizes the growth and harvest of hyperaccumulator plants that absorb high concentrations of metals in their shoots, allowing for the removal of metals from contaminated soils [97]. Despite the toxic effects of excessive potentially toxic elements in soil on plant health and other adverse impacts, hyperaccumulator plants can accumulate and tolerate high concentrations of potentially toxic elements in their living shoots [98]. Unlike phytoextraction, phytostabilization reduces the mobility of contaminants in the soil environment, thereby enabling plants to either absorb contaminants through their roots or trap them in the rhizosphere [96,99]. Optimal phytoremediation species are selected based on four key criteria: accelerated growth rates to facilitate deployment in high-contamination areas; substantial biomass production to augment contaminant uptake capacity; developed root systems to enhance contaminant accessibility; and a broad tolerance spectrum for REEs with hyperaccumulation potential in REE-contaminated environments [100,101]. Plants that accumulate REEs can extract or immobilize these elements in the soil (Table 1).

Under normal conditions, the ratio of REE content in plants to that in soil, known as the transfer factor (TF), is typically low, thereby limiting the translocation of REEs from soil to plant tissues [54]. Additionally, the transfer of REEs from soil to plants is influenced by specific parameters such as pH, soil clay concentration, and soil organic matter (OM) content [115]. In plants, the concentrations of REEs are generally low and are not essential to their physiological functions [116]. Depending on the plant species and the metal, there is a significant variation in the distribution of REEs in the main components of plants. But the concentration of REEs in underground parts is generally greater than that in shoots [7,105]. A certain level of REEs may be beneficial to plant growth and productivity, but their physiological mechanisms are not well understood, leading to recent widespread interest in the physiological and ecophysiological mechanisms underlying their responses [26]. Excessive La concentrations can affect cell division, DNA structure, nutrient absorption and photosynthesis, and induce toxic symptoms. Plants can accumulate a certain amount of La through detoxification mechanisms such as vacuole isolation, penetrant synthesis and antioxidant defense systems [48].

Significant interspecies variation in REE accumulation patterns is observed across plant taxa and organ systems, with vascular plant shoot systems consistently having negligible REE concentrations. Accurate quantification of REE concentrations in vascular plant organ systems remains methodologically constrained, particularly regarding baseline variation establishment, due to persistent challenges in isolating rhizosphere-associated REE particulates [105,117]. Vegetables like cabbage have been reported to have very low REE concentrations [118]. The distribution of REEs in the roots, stems, and leaves of vascular plants varies greatly. Comparative organ analysis reveals elevated REE concentrations being preferentially retained within root systems, which is potentially attributable to restricted translocation capacity rather than physiological transfer limitations to aerial plant structures. Experimental analyses have documented differential La accumulation between root and shoot tissues in soybeans, corn, and mung beans, with root systems exhibiting preferential retention [116,119]. Geographically distinct REE distribution patterns are observed in plant organs, with Xinjiang specimens exhibiting the distribution pattern of leaves > flowers > roots > stem concentrations versus Yunnan’s leaves exhibiting a pattern of roots > stems > flowers hierarchy, consistently demonstrating foliar dominance across phytostructural gradients [120]. Higher REE concentrations have been found in fern biomass [121], though they are comparatively lower than non-REE potentially toxic element accumulations within these botanical systems [122].

Contrary to this phenomenon, it is well known that some ferns can effectively absorb and store REEs. The remarkable accumulation of La and Ce was predominantly observed in diverse fern genera, including *Polystichum* and *Dryopteris* (Dryopteridaceae), *Diplazium* (Woodsiaceae), and *Asplenium* (Aspleniaceae) [123]. *Dicranopteris linearis* is recognized as a hyperaccumulator of REEs, with foliar concentrations typically ranging between 2 and 3 mg/g under natural growth conditions [124]. REEs are also present in trees, with observed REE concentrations and accumulation patterns in *Salix* being as follows: roots > stems > leaves. In *citrus*, the order of content distribution is roots > leaves > stems [125,126]. These advancements have laid a crucial theoretical foundation for plant-based precise remediation technology pathways targeting REE-contaminated soils.

### 5.2. Remediation of REE-Polluted Soil by Microorganisms

Microbial remediation is mediated through microbial catabolic processes that enable contaminant transformation in soil systems [127]. Microbial remediation enables in situ soil treatment, eliminating excavation requirement and minimizing the cross-contamination risk during pollutant transport [12]. This process mediates organic matter mineralization and facilitates nutrient cycling, thereby enhancing soil fertility. Microbial consortia demonstrate versatility in degrading recalcitrant organic pollutants and immobilizing heavy metal(loid)s, concurrently addressing mixed contaminants and demonstrating broad applicability across diverse pedological matrices [128].

Microbial remediation efficacy is constrained by inherent genetic instability and mutation propensity in microbial consortia. Suboptimal metabolic rates necessitate prolonged intervention periods, typically achieving partial rather than complete contaminant removal. Remediation performance is further modulated by edaphic parameters including soil temperature, moisture gradient, pH fluctuation, and oxygen availability, all of which are strict environmental optimization factors for maximal effectiveness [10]. Microbial tolerance to heavy metals is generally maintained within a specific range, but extreme deviations from this range have a negative impact on microbial growth, subsequently reducing their capacity for heavy metal adsorption and accumulation [129]. Bioremediation efficacy in contaminated soils can be optimized by conditioning the soil environment to enhance microbial viability. This biostimulation strategy requires exogenous nutrient supplementation (i.e., C/N-rich organic amendment) to elevate soil nutrient bioavailability, which augments microbial biomass and metabolic activity, thereby elevating contaminant transformation efficiency. Effective remediation microorganisms typically exhibit micrometric cellular dimensions, accelerated proliferation rates, robust metabolic pathways, and broad environmental adaptability. These traits are particularly advantageous for the remediation of REE-contaminated soils [130]. Microbial species that can accumulate REEs are cataloged in Table 2.

**Figure 3:**
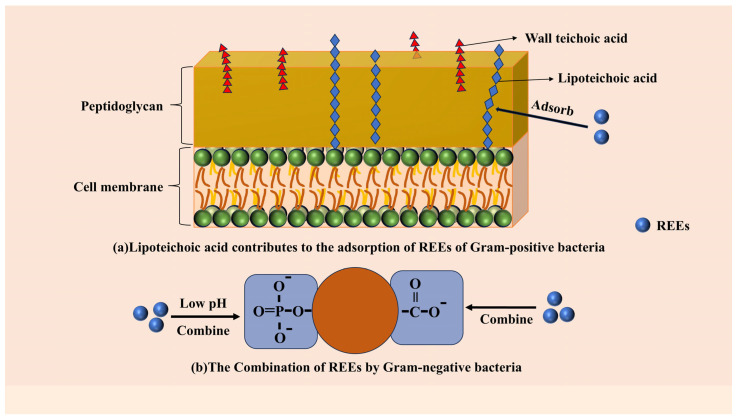
The mechanism of REE uptake by microorganisms. (**a**) Lipoyeichoic acid contributes to the adsorption of REEs of Gram-positive bacteria. (**b**) The Combination of REEs by Gram-negative bacteria.

A variety of microorganisms in soil exhibit a strong capacity for adsorbing rare-earth ions [72,73,140]. The effects of *Claroideoglomus etunicatum* on plant growth and metal absorption through interaction with soil were evaluated through greenhouse pot experiments. It was found that this system significantly increased the biomass of corn, reduced the metal concentration in contaminated soil, changed the structure of the bacterial and fungal communities in the maize rhizosphere, and regulated the abundance of the microbial quorum sensing system and metal membrane transport protein genes, therefore improving the stability and adaptability of the rhizosphere microbial community [141]. The adsorption of REEs onto the cell wall of *Bacillus subtilis* is mediated through lipoteichoic acid interactions (Figure 3a) [142]. Microbial cell surface biopolymers, including polysaccharides, glycoproteins and lipids, are functionalized with reactive binding groups (carboxyl, amino, thiol, phosphate, hydroxyl) [143]. Phosphate-mediated REE binding predominates in Gram-negative bacteria under acidic conditions, whereas carboxylate coordination becomes progressively predominant as pH levels rise (Figure 3b) [144]. From a stoichiometric perspective, inner-sphere complexation involving REEs is most likely the predominant reaction responsible for proton exchange [142].

REE–phosphate mineral interactions are enhanced by rhizospheric and microbial activities, facilitating oxalate species formation [145], and subsequently forming complexes with REEs under weakly acidic conditions [146]. Actinobacteria-mediated REE mobilization occurs through organic acid and siderophore excretion, effectively leaching REEs from bastnaesite matrices [147]. *Arthrobacter* has specific adsorption capacity for the Ho-Lu series REEs [148,149]. Microalgae can be utilized for the recovery of REEs from various sources, including ores, electronic wastes, and industrial wastewaters [150]. *Beijerinckiaceae* RH AL1 and *Methylorubrum extorquens* AM1 exhibit periplasmic lanthanide accumulation, with RH AL1 in particular storing REEs in periplasmic compartments [151]. The biosorption of La has been extensively investigated, with immobilization capabilities demonstrated in *Pseudomonas* and *Myxococcus xanthus* [152,153]. Comparative analyses reveal that biofilms exhibit a 160% greater La adsorption capacity than the *Euglena mutabilis* suspension under equivalent experimental conditions [154].

Microbially mediated litter decomposition critically governs REE behavior in the soil system. Soil OM exhibits elevated cation exchange capacity through abundant anionic functional groups, preferentially sequestering REEs via adsorption–chelation mechanisms in high-OM soils [155]. REE migration dynamics in organic-rich soil are critically modulated by dissolved organic carbon concentrations and speciation profiles. REE–humic complexes demonstrate enhanced stabilization under circumneutral pH conditions [156]. Microorganisms regulate the release and immobilization of REEs in soil through biosorption (e.g., binding REEs via cell walls and extracellular polymeric substances) [157,158] and metabolic activities (e.g., secreting organic acids to dissolve minerals or altering REE valence states) [159]. Symbiotic relationships with plants (e.g., mycorrhizae) enhance REE uptake, while environmental factors (pH, OM) and microbial community dynamics further modulate their availability. These mechanisms collectively drive REE mobility, transformation, and ecological impacts, offering biotechnological potential for pollution remediation and resource recovery.

### 5.3. Remediation of REE-Polluted Soil Using the Plant–Microorganism Combined Method

Synergistic phyto-microbial remediation systems enhance contaminant removal efficiency compared to the mono-remediation approach, helping accelerate site decontamination through complementary biochemical pathways [160]. Plant growth-promoting rhizobacteria (PGPR) are capable of metal mobilization and phytohormone production, thereby enhancing REE phytoextraction efficiency through improved nutrient bioavailability [112]. Mycorrhizal symbioses optimize rhizospheric conditions by augmenting nutrient/water acquisition, stabilizing soil microenvironments, and conferring resistance against biotic/abiotic stressors [161]. Fungal metabolites like hartzianic acid exhibit dual functionality—stimulating plant growth while demonstrating antimicrobial properties [162]. Microbial consortia concurrently enhance phytoremediation through pollutant bioavailability modulation and stress tolerance induction via secondary metabolites [163]. Arbuscular mycorrhizal fungi (AMF) demonstrate pollutant-sensitive symbiotic relationships with >80% of terrestrial plants [164], effectively mitigating La phytotoxicity while improving host water/nutrient assimilation. AMF–plant interactions mediate critical pedogenic processes by soil pore structure modification, aggregate stability enhancement, and organic–mineral complex formation [165,166,167].

The pot experiment by Guo et al. demonstrated that the symbiotic colonization of *Sorghum bicolor* roots by *Glomus versiforme* significantly enhanced La and Ce accumulation, with root concentrations exhibiting a 70% elevation compared to non-mycorrhizal controls [168]. AMF colonization not only enhanced the uptake of essential minerals (e.g., Ca, K, Zn, and N) under both control and Eu stress conditions but also mitigated Eu-induced oxidative damage, likely through the nutrient-mediated activation of antioxidant defense systems [169]. AMF can significantly mitigate Nd-induced damage and enhance the resilience of plants (*Sorghum bicolor*, *Avena sativa*) through enhanced metabolic adjustments [170]. Geochemical analyses of *Fagus sylvatica* ectomycorrhizae in southern Swedish Podzols revealed that Yb and Lu concentrations decreased within the light/intermediate REE fraction compared to La/Ce in the light fraction and Gd/Tb in the medium fraction [171]. Morrison et al. found that La–Adenosine Triphosphate complexes inhibit yeast hexokinase activity, with potency inversely proportional to ionic radii (REE^3+^ < Mg^2+^ < Mn^2+^), showing a size-dependent metalloenzyme interference pattern [172]. Previous research has compared the adsorption behavior of rare-earth metal ions using the freeze-dried powder of genetically engineered microbial strains, indicating that the three strains (*B. subtilis* 168 lipoteichoic acid-defective, wall teichoic acid-defective, and cell wall hydrolase-defective strains) have specific adsorption behavior for rare-earth metal ions [173].

As shown in Table 3, the synergistic plant–microbe remediation systems demonstrate enhanced REE removal efficiency through complementary biochemical pathways, as evidenced by PGPR-mediated metal mobilization, AMF-driven nutrient assimilation, and fungal metabolite-aided stress tolerance. However, although the tripartite interactions among REEs, soil microbiota, and plant roots are crucial for pedoecological integrity and remediation efficiency, their characterization remains insufficient. Further elucidation of these interdependencies is essential to optimize agroecosystem resilience and develop sustainable REE-polluted soil remediation frameworks.

## 6. Conclusions and Future Prospects

Contamination by REEs and potentially toxic elements in soil ecosystems results from multiple industrial activities, including but not limited to mining operations, metal extraction and smelting processes, and petroleum-refining practices, as well as emissions from medical facilities [9]. Such multi-source pollution has cumulatively posed significant threats to environmental security and human health. Conventional mono-remediation approaches, i.e., standalone phytoremediation or microbial remediation, exhibit critical limitations, including low efficiency, prolonged treatment periods, and poor field adaptability. In contrast, plant–microorganism combined remediation technology demonstrates remarkable advantages through a rhizosphere–microbe–pollutant tripartite synergy; that is, root exudates and microbial metabolites collaboratively enhance REE bioavailability [112,177], while mycorrhizal symbioses improve contaminant immobilization and nutrient acquisition [165,166,167]. Although laboratory validations have achieved some breakthroughs (i.e., a 70% increase in La/Ce accumulation in *Sorghum* roots [168]), field applications remain constrained by technical bottlenecks such as extended plant growth cycle, microbial community instability, and complex interaction in multi-contaminant systems.

Therefore, future research should prioritize studying this topic with the following objectives:

(i) To establish screening criteria for REE hyperaccumulators and decipher key mechanisms, including heavy metal ATPase transporter functions and mycorrhizal interaction dynamics (i.e., Yb/Lu migration pattern in *Fagus sylvatica* [171]);
(ii) To develop genetically engineered bacteria and a nanomaterial-mediated targeted remediation system to enhance field applicability;
(iii) To quantify the environmental risks of secondary metabolites and establish REE recovery–biomass valorization chains (pyrolysis energy density > 20 MJ/kg).
(iv) To conduct multi-site validation trials and formulate standard protocols for biomass disposal and remediation efficacy evaluation (aligned with GB 15618-2018 [178] and ISO 14040 frameworks [179]).

Through interdisciplinary integration and technological innovation, plant–microorganism combined remediation becomes a promising solution for REE-contaminated soil management, driving synergistic progress in ecological restoration and sustainable resource utilization in REE-polluted areas.

## Figures and Tables

**Table 1:** Plants that accumulate REEs.

Plants	REEs	Remediation Mechanisms	References
*Brachythecium campestr* *e* *Ceratodon purpureus*	La, Ce, Nd, Sm, Yb	Rhizofiltration	Sergeeva et al., 2021 [102]
*Cakile maritim* *Brassica juncea*	La	Phytoextraction	Bouslimi et al., 2023 [103]
*Carya illinoinensis*	Ce, La, Nd, Y	Phytoextraction	Wood et al., 2011 [104]
*Carlina corymbosa* *Erica australis* *Lavandula luisierra*	La, Ce	Phytostabilization	Anawar et al., 2012 [105]
*Dicranopteris dichotoma* *Dicranopteris linearis* *Dicranopteris pedata*	La, Ce, Pr, Nd, Sm, Eu	Phytoextraction	Chen et al., 2022 [106] Liu et al., 2021 [107] Lin et al., 2024 [108]
*Helicia australasica*	Y, Ce, Dy, Er, Tm, Yb, Lu	Phytostabilization	van der Ent et al., 2023 [109]
*Phytolacca Americana* L.	Ce, Y	Phytoextraction	Ming et al., 2018 [110] Liu et al., 2023 [111]
*Pronephrium triphyllum*	La, Ce, Nd, Sm, Eu, Tb, Yb and Lu	Rhizofiltration	Jalali et al., 2021 [112]
Rice	Nd, Dy, La	Phytoextraction	Sun et al., 2022 [56]
*Salt marshes*	Y, La	Phytostabilization	Brito et al., 2020 [113] Brito et al., 2021 [114]

**Table 2:** Microorganisms that accumulate REEs.

Microorganisms	REEs	Results	References
*Aspergillus niger*	Ce, La, Nd	The REE oxalates are precipitated by metabolites produced during fungal growth and adsorbed onto the mycelium.	Castro et al., 2020 [131]
*Mycobacterium smegmatis*	La, Sc, Y	The formation of REE (La, Sc, and Y)–siderophore complexes was observed in *Mycobacterium smegmatis*.	Andres et al., 1991 [112,132]
*Bacillus subtilis* *Saccharomyces cerevisiae* *Pseudomonas aeruginosa* *Ralstonia metallidurans* *Mycobacterium smegmatis*	Gd	The study determined the binding affinity and maximum biosorption capacity of Gd^3+^, ranging from 350 μmol/g in *Bacillus subtilis* to 5.1 μmol/g in *Saccharomyces cerevisiae*.	Andres et al., 2000 [133]
*Pseudomonas* *a* *eruginosa*	La, Eu, Yb	Under conditions of pH 5.0 and a flow rate of 0.76 m/h, the removal capacities for lanthanide cations (2 mmol/L) were 198 μmol/g for La^3+^, 167 μmol/g for Eu^3+^, and 192 μmol/g for Yb^3+^ (±10%).	Texier et al., 2000 [134]
*Bacillus cereus*	Ce, Nd	Ce and Nd demonstrated significant cellular accumulation in the two experimental groups, with dry weight accumulation levels reaching 3.02 μmol/g (Ce) and 1.40 μmol/g (Nd) in the first group, and further increasing to 7.05 μmol/g (Ce) and 3.17 μmol/g (Nd) in the second group.	Challarj et al., 2011 [135]
*Lysinbacillus* sp. DW018	Tb	DW018 can recover Tb^3+^ from wastewater, achieving a recovery rate as high as 98.28% after 10 min of treatment.	Bian et al., 2024 [136]
*Arthrobacter luteolus*	Sc, Sm	The bacterial cells exhibit a high uptake rate for LREEs, such as Sm and Sc.	Emmanuel et al., 2012 [137]
*Bacillus* sp. Z2	Y	It can effectively reduce the bioavailability of Y^3+^.	Wang et al., 2023 [138]
*Galdieria sulphuraria*	Nd, Dy, La	The algae achieved over 90% recovery efficiency for Nd (III), Dy (III), and La (III) at a concentration of 0.5 ppm, and maintained stability within a pH range of 1.5–2.5.	Minoda et al., 2015 [139]

**Table 3:** Joint effects of plants and microorganisms on REEs.

Plants	Microorganisms	Outcomes of Combined Effects	References
Rice	*Anaeromyces Obacter* *Georgfuchsia*	These rhizospheric bacteria can significantly increase the content of REEs in rice, making it notably higher than that in soil from non-rare-earth mining areas.	Zhang et al., 2022 [45]
*Helianthus annuus*	*Bacillus* sp.	The combined system significantly enhanced the phytoextraction capacity of REEs, with the concentrations of Ce, La, Nd, and Y reaching 4.4, 38.3, 3.4, and 21 times those of the control group, respectively.	Jalali et al., 2021 [112]
*Sorghum bicolor* L.	*Glomus versiforme*	The concentrations of La, Ce, Pr, and Nd in the roots of sorghum increased by approximately 70%.	Guo et al., 2013 [168]
*Phytolacca americana*	*Rhodanobacter*	*Rhodanobacter* promotes the growth of *Phytolacca americana*, thereby enhancing the phytoremediation efficiency of *Phytolacca americana* for REEs.	Yan et al., 2024 [174]
*Buffalo grass*	*Rhizophagus*	The activity of arbuscular mycorrhizae typically increases the mass of REEs in plant tissues by a factor of 1.2 to 1.6.	Zaharescu et al., 2017 [175]
*Neobuxbaumia macrocephala*	*Actinobacteria* *Sphingobacteriia*	The bacteria isolated from the surface, rhizosphere, and stems of this plant exhibit metabolic characteristics dependent on REEs (Ce).	del Rocío et al., 2017 [176]

## Data Availability

Data available on request due to restrictions.
